# Hospital Meetings

**Published:** 1906-04-07

**Authors:** 


### HOSPITAL MEETINGS.

### NATIONAL HOSPITAL FOR THE PARALYSED AND EPILEPTIC.

The chair at the annual meeting of Governors on March 27 was taken by Mr. Frederick Macmillan, Chairman of the Board of Management.

The adoption of the report was moved by the Chairman, who said that the year's work was on the whole satisfactory. The general spirit pervading the management and staff of the hospital was anxiety that everything should be kept in the very best state of efficiency, and that no pains should be spared to bring about this result. The important work done by the medical and surgical staff was far-reaching in its results. The effects of it were felt, not only by the in- and out-patients, but indirectly by the great number of those suffering from nervous diseases, who benefited through the experience gained at this hospital. While they were determined to strive for efficiency, they were equally determined to strive for economy; the two should go hand in hand, for where loose methods of doing things prevailed the result was extravagance. They were following the economical lines laid down when the present Board first took office. The spirit of enconomy then started still animated all departments of the hospital. It was only a few months ago that their secretary received the prize for the best essay on the economical management of an efficient voluntary hospital, which showed that they had the best advice. The affairs of the hospital were looked into with great care by the secretaries of King Edward's Hospital Fund and the Hospital Sunday Fund. He was glad to welcome there that day one of the secretaries of King Edward's Fund. Important work was being done by the Nervous Diseases Research Department, which had been put on a satisfactory basis during the last year or two. They had had the misfortune to lose the services of Miss Vernet, who, after doing most efficient work as Matron for the last seven years, had gone to be Matron at the Middlesex Hospital. Her place had been filled by Miss Lawrence, whom they were glad to think had already spent many years in the hospital. The finances were no worse this year than they had been before, but at the same time they had a very anxious year before them, as they did not feel able to make any special appeal this year, such as the festival they had had last year. A circular had been sent round to the Governors pointing out the importance of increasing the annual subscriptions, which was the only form of income on which they could rely.

The resolution was seconded by Mr. J. Danvers Power, who said he noticed in the report two facts which showed the admirable working of the hospital; one was that only five out of the 160 beds had not been continuously occupied during the year. This showed the great demand there was upon the beds in this hospital and the excellent use made of them, thus preventing any waste. The other fact was that the average cost per bed was £87 8s. 11d. As Hon. Secretary of King Edward's Hospital Fund he had that day been asked by a very rich man whether a certain hospital was economically carried on, and he had been able to assure him it was, though the average cost per bed was £8 more than that at the National Hospital for the Paralysed and Epileptic. The ladies' committee had done good and lasting service, it had effected an immediate decrease in expenses, a decrease which was progressive. After referring to the promotions of some members of the staff and to the essay written by the secretary, which was absolutely the best sent in, and had won the prize in an open field, he went on to speak of the school of massage, the profits from which had been £107. He strongly advised more advertising being done on behalf of this school and a greater effort being made to provide employment for those who made use of it.

The Chairman remarked that against the profits of this school must be set the increase in the nursing salaries.

Sir John Paget proposed the adoption of the statement of receipts and expenditure. The total ordinary income was £13,517, and the ordinary expenditure £15,709. The deficit was amply covered by what they had received in extraordinary income. They had been very fortunate in legacies, which amounted to £8,424, and they had thus been enabled to pay off £4,583 of their debt to their bankers. They had received several sums for the endowment fund, the interest upon which they would be able to add to their income. They had a loss on their convalescent home, but this could not be avoided, and they had been obliged to make it a grant from the general fund.

The accounts were passed, and Dr. Beevor later proposed Lord Strathcona and Mount Royal and Mr. J. Danvers Power as vice-presidents. This was passed, as was also Sir Felix Semon's resolution of a vote of thanks to the board and all the staff of the hospital.

### NORTH-WEST LONDON HOSPITAL.

The annual general Court of Governors was held at the hospital on March 29th, when the chair was taken by Lord Rathmore.

In proposing the adoption of the annual report, Lord Rathmore said that it showed how much good had been done, and how much suffering relieved by a small and unostentatious hospital. The in-patients had numbered 631 and out-patients 23,324, including 7,052 casualties. The total number of out-patients' attendances was 44,699. The income from all sources amounted to £4,272, which included what was very satisfactory, the sums of £750 from King Edward's Hospital Fund, £629 from the Hospital Sunday Fund, and £146 from the Hospital Saturday Fund. The fact that those who had the distribution of these charitable funds felt justified in each case in increasing the amount awarded to this hospital proved how well they appreciated the care and zeal with which the managers and staff applied the slender funds at their disposal. After referring with gratitude to the action of Mr. B. Pearce Lucas in placing the New Bedford Palace of Varieties at the disposal of the hospital, whereby the sum of £251 was raised by means of a matinée performance, he mentioned that among the numerous gifts to the hospital was a present of game from the King. The success of the hospital was due to the unflagging zeal with which the staff discharged their duties. He also wished specially to mention the great benefits conferred by Mr. George Herring's soup kitchen, which had proved an inestimable boon to the destitute poor of that crowded neighbourhood. Mr. Herring was not only a generous benefactor of the hospital in many ways, but he also found time from his many important business and philanthropic engagements to come there week after week and give the committee the great advantage of his experience and advice.

With regard to the need for a larger and more commodious building, if only the money could be obtained, it would be an enormous benefit to that neighbourhood if the hospital could be rebuilt and enlarged. At present they had 50 beds available, but, as the house surgeon had that day told him, if they had 150 beds available it would be easy to fill them all. They could only appeal once more to the generosity of the public and urge them to come to their assistance, and by larger contributions and benefactions enable them to maintain and expand the merciful and useful work that hospital continued to carry on with very slender means.

The resolution, having been seconded by Mr. Bangs, was adopted.

Dr. Stowers, in responding to a vote of thanks to the staff for the splendid way in which they carried on their work under exceptionally difficult circumstances, declared that if they had 250 beds available in that hospital they would not have ten empty. For want of special apparatus patients requiring special treatment had to be sent to other hospitals; only that afternoon he had been compelled to recommend two patients to go to other hospitals. The work of that hospital would be carried on even better than it was if only they could obtain this special apparatus, so essential to the treatment of certain diseases. He felt sure if the needs of that institution were more widely known they would be able to obtain more funds.

A vote of thanks to the Chairman brought the proceedings to a close.

### HOSPITAL FOR EPILEPSY AND PARALYSIS.

Mr. G. Knapp Fisher presided at the annual Court of Governors of this hospital, held on March 31. The adoption of the report and balance sheet was moved by the chairman, who said that the report was a simple record of everyday work done in a humdrum way. There was one statement in the report which was a little misleading; that was the one which spoke of this being the first year during which the completed part of the hospital had been in working with the full number of 40 beds. Anyone who had seen the hospital from outside would have noticed that it was as yet in a far from completed condition, and they wanted a large sum of money before they could finish it, which as yet there was no prospect of their obtaining. At present they had 40 beds, but when completed they would have from 60 to 70. It was very pleasing to the committee to think that they had been able to raise the money needed for the first portion and open the building absolutely free of debt. It was now 22 years since he had joined the committee of the hospital, and he observed that Dr. Ogilvie and Captain Ellis were the only members of the committee who had been on it at that time He had been drawing comparisons between the position of the hospital 22 years ago, and at the present time and he did not find the comparisons altogether pleasing. The expenditure 22 years ago was £2,400; it was now £3,000. Considering that the hospital was then very much smaller, with only 23 beds and a much smaller staff, whereas they now had 40 beds, which necessitated a large increase in expenses, it was very gratifying to find the expenditure had only increased by £600. This showed that the expenditure had been reduced to a minimum. But when he came to look at the receipts it was a different story. The annual subscriptions for 1905 were only £316, which he called a miserable sum. Twenty-two years ago they were £366. Donations now were £312; then they were £873. The in-patients' payments now were £418; then they were £521. The out-patients' payments now were £221; then they were £250, and this notwithstanding the fact that the number of patients had increased enormously. In 1905 they had had 166 in-patients, with an average rate of stay of 75 days; 22 years ago the number was 141, and the length of stay 54 days; so that there was a large increase both in the number of in-patients and in the duration of their stay at the hospital. The attendances of out-patients were nearly 13,000 in 1905, and yet their payments were only £221; whilst with 7,688 attendances 22 years ago their payments were a little more. It was difficult to say what this state of things was due to, partly perhaps to the wave of pauperism that seemed to be sweeping over the land, and which affected them as a hospital as it affected everything. If it had not been for the grant from King Edward's Hospital Fund, and for the sale of some invested property which had been put by in better times, he did not know what the committee would have done. As an instance he mentioned that on December 31, 1904, they had £504 in hand; on December 31, 1905, they had only £134. Unless they could obtain some very substantial aid shortly, they would have nothing to do but close part of the hospital. It was impossible to go on with an increasing expenditure and decreasing receipts. He did not consider people did what they ought to do for the support of the hospitals; they were here in the midst of very rich neighbours, who should come to their aid. He appealed to them to ask those who could not give guineas to give half-guineas, or take a collecting-box, or to do something for the support of a hospital which was so economically and well worked.

Dr. Ogilvie seconded the motion, and after votes of thanks had been passed to the staff and to the chairman for presiding, the proceedings terminated.

*** We regret that owing to pressure on our space the reports of certain hospital meetings have been unavoidably held over until next week.

